# Volatile Anesthetics, Not Intravenous Anesthetic Propofol Bind to and Attenuate the Activation of Platelet Receptor Integrin αIIbβ3

**DOI:** 10.1371/journal.pone.0060415

**Published:** 2013-04-03

**Authors:** Koichi Yuki, Weiming Bu, Motomu Shimaoka, Roderic Eckenhoff

**Affiliations:** 1 Department of Anesthesiology, Perioperative and Pain Medicine, Boston Children's Hospital, Boston, Massachusetts, United States of America; 2 Department of Anesthesiology and Critical Care, Perelman School of Medicine, University of Pennsylvania, Philadelphia, Pennsylvania, United States of America; 3 Department of Molecular Pathobiology and Cell Adhesion Biology, Mie University, Graduate School of Medicine, Tsu, Mie, Japan; University of Colorado, United States of America

## Abstract

**Background:**

In clinical reports, the usage of isoflurane and sevoflurane was associated with more surgical field bleeding in endoscopic sinus surgeries as compared to propofol. The activation of platelet receptor αIIbβ3 is a crucial event for platelet aggregation and clot stability. Here we studied the effect of isoflurane, sevoflurane, and propofol on the activation of αIIbβ3.

**Methods:**

The effect of anesthetics on the activation of αIIbβ3 was probed using the activation sensitive antibody PAC-1 in both cell-based (platelets and αIIbβ3 transfectants) and cell-free assays. The binding sites of isoflurane on αIIbβ3 were explored using photoactivatable isoflurane (azi-isoflurane). The functional implication of revealed isoflurane binding sites were studied using alanine-scanning mutagenesis.

**Results:**

Isoflurane and sevoflurane diminished the binding of PAC-1 to wild-type αIIbβ3 transfectants, but not to the high-affinity mutant, β3-N305T. Both anesthetics also impaired PAC-1 binding in a cell-free assay. In contrast, propofol did not affect the activation of αIIbβ3. Residues adducted by azi-isoflurane were near the calcium binding site (an important regulatory site termed SyMBS) just outside of the ligand binding site. The mutagenesis experiments demonstrated that these adducted residues were important in regulating integrin activation.

**Conclusions:**

Isoflurane and sevoflurane, but not propofol, impaired the activation of αIIbβ3. Azi-isoflurane binds to the regulatory site of integrin αIIbβ3, thereby suggesting that isoflurane blocks ligand binding of αIIbβ3 in not a competitive, but an allosteric manner.

## Introduction

General anesthesia during surgery is induced and maintained by administration of inhalational (volatile) and/or intravenous anesthetic drugs. While anesthetic drugs primarily act on neuronal cells in the central nervous system [1], thereby inducing general anesthetic states, the report that halothane impairs adenosine diphosphate (ADP)-induced platelet aggregation by Ueda [2] triggered subsequent studies on the effect of hemostasis. Clinical observational investigations into the effects of anesthetics on hemostasis during surgery [3], point to an intriguing trend that intra-operative bleedings were less severe in anesthesia with the intravenous anesthetic propofol than volatile anesthetics isoflurane and sevoflurane [3], [4], [5], [9], [10] (**Table 1**). However, *in vitro* mechanistic investigations into the direct effects of propofol [12], [13], isoflurane [10], , and sevoflurane [12], [14] [16] on platelet aggregation, a critical step in hemostasis have shown mixed results thus far.

**Table 1:** The effect of anesthetics on surgical bleeding.

Surgical procedure	Anesthetics and number of patients	Study design	Results	Refer-ence
Endoscopic sinus surgery	Sevoflurane/remifentanil (n = 20) versus propofol/remifentanil (n = 20)	Prospective, randomized study	Less blood loss and better surgical field in propofol group for patients with extensive chronic sinusitis	[3]
Endoscopic sinus surgery	Propofol (n = 30) versus isoflurane (n = 26)	Prospective, randomized study	Better surgical field	[8]
Endoscopic sinus surgery	Propofol (n = 12) versus isoflurane (n = 13)	Retrospective review	Decreased blood loss in propofol group	[4]
Endoscopic sinus surgery	Propofol/remifentanil (n = 45) versus isoflurane/alfentanil (n = 43)	Prospective, randomized study	Bleeding from surgical field was significantly better in propofol group	[5]
Endoscopic sinus-nasal surgery	Propofol/remifentanil (n = 27) versus isoflurane/fentanyl (n = 37)	Prospective, randomized study	Propofol/remifentanil was effective in reducing bleeding	[10]
Endoscopic sinus surgery	Sufentanil/Sevoflurane (n = 23) versus remifentanil/propofol (n = 20) versus fentanyl/isoflurane (n = 28)	Retrospective review	Least bleeding in remifentanil/propofol group	[7]
Endoscopic sinus surgery	Sevoflurane/fentanyl (n = 28) versus propofol/remifentanil (n = 28)	Prospective, randomized study	Better surgical field in propofol/remifentanil group	[11]
Endoscopic sinus surgery	Propofol/fentanyl (n = 16) versus sevoflurane/fentanyl (n = 16)	Prospective, randomized study	Less bleeding in propofol group	[9]
Head and neck surgery	Isoflurane (n = 20) versus propofol (n = 18)	Prospective, randomized	Blood loss in isoflurane group tended to be slightly higher.	[6]

**Figure 1:**
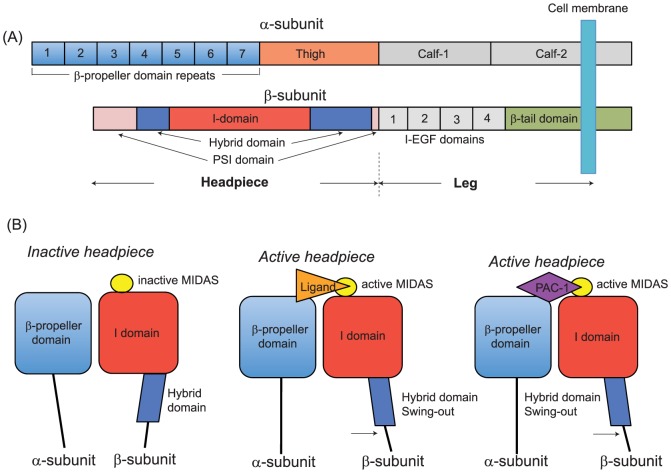
Integrin structure and conformational change. (A) αIIbβ3 consists of the α subunit (αIIb) and the βsubunit (β3). Domains within the primary structure of α- and β- subunits suggested by X ray crystal structures of αVβ3 and αIIbβ3 [24], [52] are shown. The β-propeller and the thigh domains of the α subunit and the PSI, the hybrid and the I domains of the β subunit constitute the headpiece of αIIbβ3. (B) Schema of conformational change of the headpiece. The metal-ion dependent adhesion site (MIDAS) undergoes conformation change and interacts directly with ligands when it is in an active form. In a conformation where the hybrid domain faces inward toward the α subunit, the MIDAS is inactive. When the hybrid domain swings out, the conformational change of the MIDAS ensues with ligand or PAC-1 binding.

αIIbβ3 is the most abundant receptor in platelets and plays a critical role in platelet aggregation and clot stability through the interaction with its Arg-Gly-Asp (RGD)-motif –containing ligands fibrinogen, von Willebrand factor and fibronectin [17], [18], [19], [20], [21], [22]. αIIbβ3 is a member of the adhesion molecule family integrins, and is composed of non-covalently linked α/β heterodimers, with each subunit consisting of multiple well-characterized domains [23](**Figure 1A**). Only upon activation, αIIbβ3 undergoes the conformational changes referred to as “the hybrid domain swing-out”, which induces the ligand binding site to the high-affinity state [24] (**Figure 1B**). Three metal binding sites (metal-ion dependent adhesion site (MIDAS), SyMBS, and ADMIDAS) located on the top of the β3 I domain differentially regulate the activity of integrin αIIbβ3 during this conformational change. The MIDAS directly binds to the RGD motif of ligands, while SyMBS and ADMIDAS take indirect roles in ligand binding by modulating metal coordinations at the MIDAS [24],[25],[26]. The study by Horn et al. demonstrated that sevoflurane, even at subanesthetic concentrations, significantly abolished the activation of αIIbβ3 in whole blood [27]. Inspired by Horn et al, and building on our previous studies on the effects of volatile anesthetics to leukocyte integrins [28],[29],[30],[31], here we tested the hypothesis that isoflurane and sevoflurane, not propofol directly interacted with platelet integrin αIIbβ3and interfered with its activation.

## Materials and Methods

### Cells

Chinese hamster ovary (CHO) -K1 cells stably transfected with αIIb-wild type (WT)/β3-WT or αIIb-WT/β3-N305T were previously described and kindly given by Dr. Springer [32]. They were cultured in RPMI1640, 10% FBS and geneticin G418 in 5% CO_2_ at 37°C. 293T cells (ATCC; Manassas, VA, USA) were cultured in DMEM with HEPES modification, 10% FBS at 37°C in 5% CO_2_.

### PAC-1 binding assay using human platelets

The activation of αIIbβ3 was probed using PAC-1, an IgM antibody that binds only to the activated αIIbβ3 [33], [34]. Freshly prepared platelet-rich plasma (PRP) was purchased from Research Blood Components, LLC (Boston, MA, USA). PRP was diluted in Tyrode's buffer (1% bovine serum albumin (BSA), 2 mmol/L MgCl_2_, 137.5 mmol/L NaCl, 12 mmol/L NaHCO_3_, 2.6 mmol/L KCl, pH 7.4) as described [35], and stimulated with 20 µM adenosine 5′- diphosphate (ADP) (Sigma; St. Louis, MO, USA) in the presence of PAC-1-FITC (BD Biosciences; San Jose, CA, USA) and anesthetics (isoflurane or propofol) for 30 minutes. Isoflurane was administered to PRP in the closed chamber using a Fluotec vaporizer (Cyprane Ltd., Keighley, UK), and their concentrations were measured using infrared spectroscopy (Datex Instrument Corp., Helsinki, Finland). Following stimulation, samples were fixed with paraformaldehyde (1%) and subject to the flow cytometry anaylsis using a FACScan (BD Biosciences; San Jose, CA, USA). Data were shown as mean fluorescence intensity (MFI).

### PAC-1 binding assay using αIIbβ3 transfectants

CHO-K1 cells transfected with αIIbβ3 were detached in HEPES-buffered saline (HBS)/10 mM EDTA and washed three times with HBS. Cells were incubated with 10 µg/ml PAC-1 (BD Biosciences) in HBS containing 1 mM MgCl_2_/CaCl_2_ (inactivating condition) or HBS Containing 1 mM MnCl_2_/0.4 mM CaCl_2_ (activating condition) in the presence of various concentrations of isoflurane, sevoflurane or propofol. Isoflurane and sevoflurane were administered to cells in the closed chamber using a Fluotec vaporizer, and their concentrations were measured using infrared spectroscopy. Goat anti-mouse IgM-FITC (Santa Cruz biotechnology Inc.; Santa Cruz, CA, USA) was used as a secondary antibody. Cells were analyzed with a FACScan. In addition, the cell surface expression of αIIbβ3 was probed with AP3 antibody (Immune Disease Institute; Boston, MA, USA). PAC-1 binding % was calculated as [(MFI of sample at various concentrations of anesthetics)-(MFI of isotype control sample)]/(MFI of sample without anesthetics)-(MFI of isotype control sample)]×100%.

### Protein expression and purification

The purification of full-length ectodomain and headpiece αIIbβ3 was previously described [36], [37]. Integrin αIIbβ3 purified from human platelets was purchased from EMD Millipore (Billerica, MA, USA).

### PAC-1 binding to the extracellular portion of αIIbβ3

Capturing antibody AP3 was coated on 96 wells overnight. Wells were blocked with 2% BSA and then incubated with recombinant αIIbβ3 (full length or headpiece). Following washing, wells were incubated with PAC-1 in the presence of various concentrations of isoflurane, sevoflurane or propofol containing 1 mM MgCl_2_/CaCl_2_ or 1 mM MnCl_2_/0.4 mM CaCl_2_ for 1 hour. Isoflurane and sevoflurane were administered to wells in a closed chamber using a Fluotec vaporizer and their concentrations were measured using infrared spectroscopy. Attached αIIbβ3 was captured with anti-mouse IgM- HRP (Cayman Chemical; Ann Arbor, Michigan, USA). Color was developed with substrate (BD Bioscience; San Jose, CA, USA). Optical density (OD) at 405 nm was read using an ELISA plate reader (Molecular Device; Sunnyvale, CA, USA). PAC-1 binding % was defined as [(OD of sample at various concentrations of anesthetics) – (OD of background)]/[(OD of sample without anesthetics) - (OD of background)]×100%.

### Photolabeling experiments

Photolabeling experiments were performed using azi-isoflurane, isoflurane with a diaryzinyl moiety. The details of experiment have been previously described [38], [39]. Briefly, full-length ectodomain αIIbβ3 or αIIbβ3 purified from human platelets was incubated with or without 1 mM azi-isoflurane in quartz cuvettes for 15 minutes, and then exposed to 300 nm UV light for 15 minutes. The protein was separated using sodium dodecyl sulfate polyacrylamide gel electrophoresis (SDS-PAGE). Bands corresponding to the protein of interest were excised, trypsinized and submitted for nano liquid chromatography (LC)/mass spectrometry (MS) analysis. LC was performed using a 10 cm C18 capillary column at 200 nl/min for 60 minutes with gradient elution. MS-detected peptides were searched for adducts of the appropriate mass (196 Da) and then further fragment patterns (MS/MS) were searched using *Sequest* software to determine the adduct attachment sites. Mass spectrometry work was performed at the University of Pennsylvania Proteomics Core Facility.

### Point mutagenesis and transfection

Alanine scanning mutagenesis was performed using Quikchange XL kit (Stratagene; La Jolla, CA, USA). DNA sequence was confirmed. Transfection was performed using Lipofectamine 2000 (Invitrogen; Carlsbad, CA, USA) per company protocol.

### Statistical significance

Data were analyzed using an analysis of variance (ANOVA) with Tukey post hoc pairwise comparisons or student's t-test as indicated in corresponding figure legends. Statistical significance was defined as P<0.05. Statistical analysis was performed using PRISM 5 software (GraphPad Software; La Jolla, CA, USA).

## Results

### Isoflurane and sevoflurane, but not propofol attenuated PAC-1 epitope exposure in ADP stimulated platelets, but propofol did not

**Figure 2:**
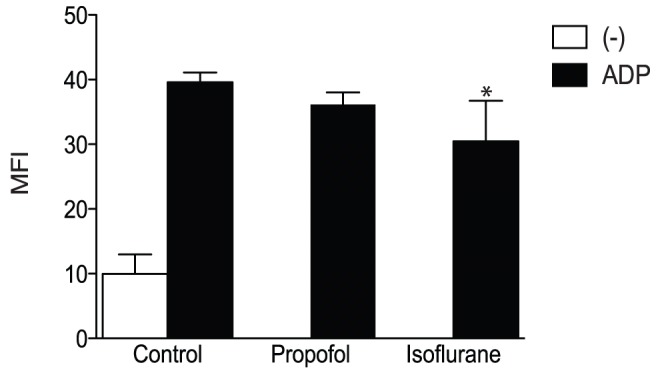
PAC-1 binding assays with anesthetics in platelets. Flow cytometry based PAC-1 binding assays were performed using platelet-rich plasma stimulated with 20 µM adenosine 5′-diphosphate (ADP) in the presence of isoflurane (2%) or propofol (50 µM). Data is shown as mean +/− S.D. of mean fluorescence intensity (MFI) of six independent experiments. Data were analyzed using a one-way analysis of variance with Tukey post hoc pairwise comparisons. * denotes *p*<0.05 versus ADP-treated control sample.

From clinical observational studies on hemostasis as summarized in **Table 1**, we hypothesized that isoflurane and sevoflurane would attenuat the activation of integrin αIIbβ3, but propofol would not. In fact, volatile anesthetic sevoflurane attenuated the activation of αIIbβ3 on platelets stimulated by ADP as demonstrated by Horn et al. [27]. We demonstrated that another volatile anesthetic isoflurane at a clinically relevant concentration (2%) attenuated its activation on platelets (**Figure 2**). The clinical relevant concentration of propofol ranges from 10–50 µM [40], [41], [42]. Propofol (50 µM) did not attenuate the activation of αIIbβ3 on platelets (**Figure 2**). These results supported our hypothesis. To assess the direct interaction of volatile anesthetics with αIIbβ3, we examined the effect of anesthetics using CHO cells stably transfected with αIIbβ3 and purified proteins in the following sections.

### Isoflurane and sevoflurane attenuated the activation of wild type αIIbβ3 on cells

**Figure 3:**
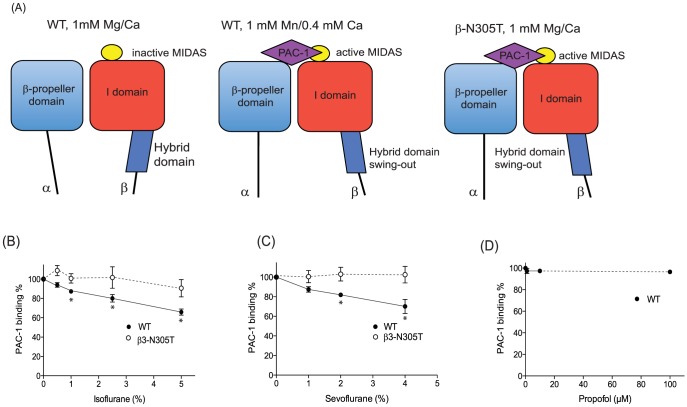
PAC-1 binding assays using αIIbβ3 transfectants in the presence of anesthetics. (A) Scheme of PAC-1 interaction with αIIbβ3 WT and β3-N305T mutant. While αIIbβ3 wild-type (WT) binds to PAC-1 only in an activating condition (1 mM Mn^2+^/0.4 mM Ca^2+^), activating β3-N305T mutant can bind to PAC-1 in a resting condition (1 mM Mg^2+^/Ca^2+^) due to its constitutive swing-out of the hybrid domain. (B–D) Flow cytometry based PAC-1 binding assays were performed using CHO cells stably transfected with wild type αIIbβ3 or N305T mutant in the presence of isoflurane (B) or sevoflurane (C) at various concentrations. For propofol, only wild type αIIbβ3 was tested (D). PAC-1 binding % was calculated as [(mean fluorescence intensity (MFI) at various concentrations of anesthetics) – (MFI of isotyoe control)]/[(MFI without anesthetics) – (MFI of isotype control)]×100 (%). Data is shown as mean +/− S.D. of three independent experiments. Binding experiment was done at 1 mM Mn^2+^/0.4 mM Ca^2+^. One-way analysis of variance with Tukey post hoc pairwise comparisons was used to compare the data at different anesthetic concentrations within wild-type or mutant transfectants. * denotes p<0.05 versus mock-treated sample (no anesthetic).

**Figure 4:**
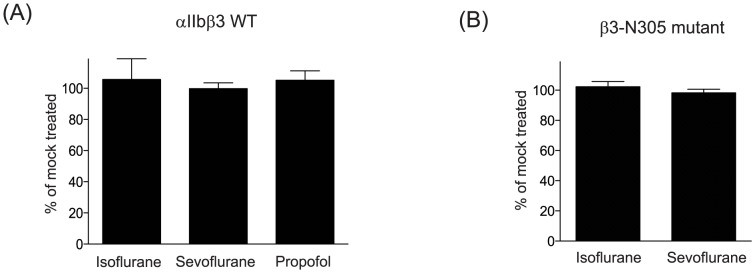
The effect of anesthetics on αIIbβ3 cell surface expression. Surface expression of αIIbβ3 WT (A) or β3-N305T (B) was probed by AP3 antibody and expressed using mean fluorescence intensity (MFI). Data was shown as [(MFI of αIIbβ3 exposed to anesthetic)/(MFI of αIIbβ3 of sample not exposed to anesthetic)]×100%, and expressed as mean +/− S.D. of three independent experiments. Isoflurane, sevoflurane, and propofol used were 5%, 4%, and 100 µM, respectively.

First, we evaluated the effect of anesthetics on αIIbβ3 activation in CHO transfectants. PAC-1 contains the Arg-Tyr-Asp (RYD) sequence that is analogous to the RGD sequence in the complementarity determining region 3 of the heavy chain. This region is speculated to interact with the activated MIDAS [33]. We tested PAC-1 binding either in a resting condition (1 mM Mg^2+^/Ca^2+^) or an activating condition (1 mM Mn^2+^/0.4 mM Ca^2+^). In 1 mM Mg^2+^/Ca^2+^, PAC-1 does not binds to αIIbβ3 WT (**Figure 3A**). On the other hand, PAC-1 binds significantly to αIIbβ3 WT in 1 mM Mn^2+^/0.4 mM Ca^2+^ (**Figure 3A**). Isoflurane and sevoflurane diminished PAC-1 binding to WT in 1 mM Mn^2+^/0.4 mM Ca^2+^ (**Figure 3B and C**), while they did not alter the expression of αIIbβ3 WT (**Figure 4A**). This suggested that isoflurane and sevoflurane either attenuated the activation of αIIbβ3 WT or directly interacted with PAC-1 binding sites. The β3-N305T mutant was previously designed to introduce N-glycosylation by changing amino acid sequences of the β3 subunit from N^303^-I^304^-N^305^ to N^303^-I^304^-T^305^ [32]. β3-Asn305 is located on the bottom of the I domain, at the interface with the hybrid domain. The introduction of N-glycan at this site opened up the interface between the I domain and the hybrid domain, mimicking the hybrid domain swing-out motion and making this mutant constitutively active [32] (**Figure 3A**). Both isoflurane and sevoflurane failed to modulate PAC-1 binding to β3-N305T mutant (**Figure 3B and C**). Further, exposure to isoflurane and sevoflurane did not alter the expression level of αIIbβ3 on the β3-N305T mutant (**Figure 4B**), suggesting that these volatile anesthetics did not directly interact with PAC-1 binding sites on αIIbβ3. Taking these results together, isoflurane and sevoflurane attenuated the activation of αIIbβ3 WT. In contrast, the intravenous anesthetic propofol failed to modulate PAC-1 binding to αIIbβ3 WT (**Figure 3D**), indicating that propofol did not inhibit the activation of αIIbβ3.

### Isoflurane and sevoflurane attenuated the activation of αIIbβ3 protein

**Figure 5:**
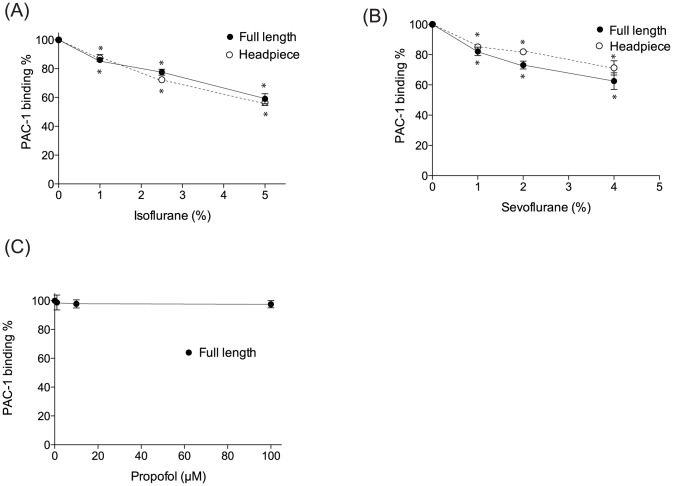
Cell-free PAC-1 binding assays with anesthetics. ELISA type PAC-1 binding assays were performed using full-length ectodomain or headpiece αIIbβ3 in the presence of isoflurane (A) or sevoflurane (B) at various concentrations. For propofol, experiments were performed using full-length αIIbβ3 (C). PAC-1 binding % was calculated as [(OD at various concentrations of anesthetics)- (OD of background)]/[(OD of mock treated sample)-(OD of background)]×100 (%). Data is shown as mean +/− S.D. of three independent experiments. Binding experiment was done at 1 mM Mn^2+^/0.4 mM Ca^2+^. One-way analysis of variance with Tukey post hoc pairwise comparisons was used to compare the data at different anesthetic concentrations within full-length or headpiece protein. * denotes p<0.05 versus mock-treated sample (no anesthetic).

We demonstrated that isoflurane and sevoflurane attenuated the activation of αIIbβ3 in cell-based assays using CHO transfectants. Anesthetics are well appreciated as promiscuous molecules [43], and thus we cannot conclude if the results reflected the direct effect of the anesthetics on αIIbβ3, or the indirect effect (for example, the effect on the plasma membrane or intracellular proteins). We examined the effect of anesthetics on the activation of purified αIIbβ3 protein in cell-free ELISA type assay, which excluded the components of the plasma membrane and intracellular proteins. Both isoflurane and sevoflurane impaired the activation of αIIbβ3 (**Figure 5A and B**). Interestingly, there was no difference in the degree of inhibition between headpiece and full-length αIIbβ3, suggesting that isoflurane and sevoflurane interacted with the headpiece portion ofαIIbβ3. Propofol did not affect the activation of αIIbβ3 in this cell-free assay (**Figure 5C**), as predicted by the previous result (**Figure 3D**).

### Azi-isoflurane bound to the βI domain

**Figure 6:**
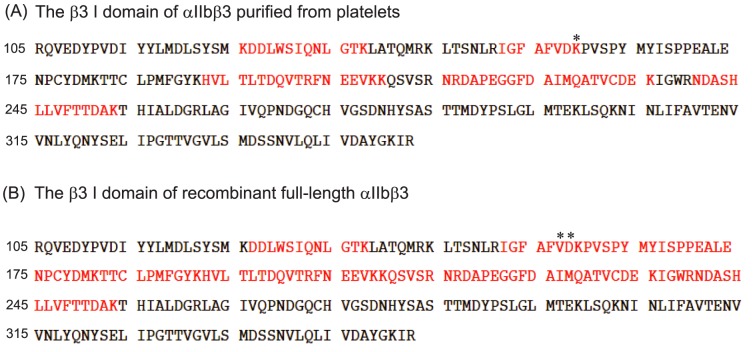
Amino acid residues of the β I domain covered by mass spectrometry. The amino acid residues of the β I domain are shown. Covered residues by mass spectrometry are shown in red. Adducted residues are shown in asterisk.

**Figure 7:**
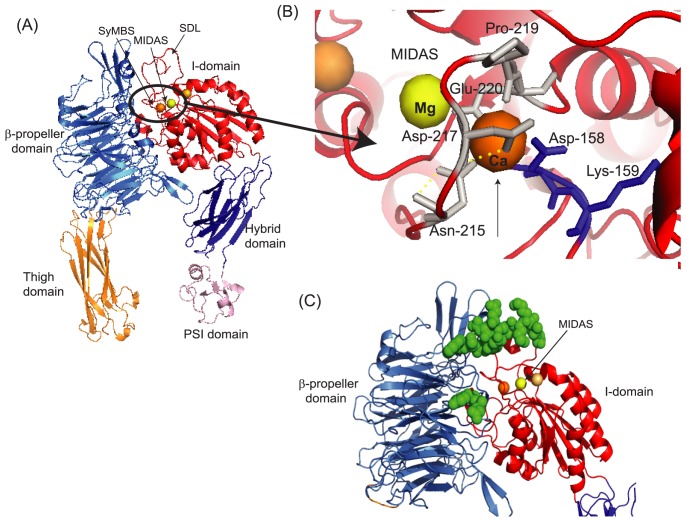
αIIbβ3 headpiece structure and adducted residues. (A) The X ray crystal structure of αIIbβ3 headpiece was obtained from protein data bank (PDB; 3FCS). There are three metal binding sites in the I domain of the β subunit. Mg^2+^ in the MIDAS (this site is directly involved in ligands binding) is shown in yellow sphere, while Ca^2+^ in the SyMBS and ADMIDAS are shown in orange and light orange spheres, respectively. (B) The blowout of residues around metal binding sites from Figure 7 (A) is shown. The adducted residues of photolabeling experiments are shown in blue. Again, Mg^2+^ in the MIDAS is shown in yellow sphere, while Ca^2+^ in the SyMBS is shown in orange sphere. Both figures were created using PYMOL. (C) The structure of αIIbβ3 in the open conformation was obtained from Protein data bank (http://www.rcsb.org/pdb/home/home.do; PDB 3FCU). Residues shown as green spheres on αIIbβ3 are suggested PAC-1 binding sites by Puzon-McLaughlin et al. [44]. This figure was created using PYMOL.

Our cell-free and cell-based assays strongly suggested that isoflurane and sevoflurane directly interacted with αIIbβ3 and attenuated its activation. Previously we reported that a novel photoactivatable compound, azi-isoflurane [38], reliably probed isoflurane binding sites on apoferritin, integrin αL I domain [39], and LFA-1 [31]. Thus, we used azi-isoflurane to reveal isoflurane binding sites on both full-length recombinant αIIbβ3 and purified αIIbβ3 from platelets. Azi-isoflurane bound to the I domain at Asp-158 and/or Lys-159 (**Table 2**, **Figure 6** **,** **7A and 7B**) in both samples. Asp-158 and Lys-159 are close to the calcium binding site as shown in **Figure 7B**. This calcium binding site is called the synergistic metal binding site (SyMBS) or the ligand associated metal binding site (LIMBS) [26]. The adducted residues were in the headpiece region of αIIbβ3, which was in line with our result of cell-free assays. The epitope mapping of PAC-1 by Puzon-McLaughlin et al. showed that they were within residues 156–162 and 229–230 of the αIIb subunit and residues 179–183 of the β3 subunit (**Figure 7C**) [44]. Our adducted residues did not belong to these residues, which suggested that volatile anesthetics did not compete with PAC-1 directly as we indicated based on the results of cell-based assays. Unfortunately, a photoactive version of sevoflurane is not currently available, and we were not able to explore the binding site of sevoflurane using this approach. However, sevoflurane and isoflurane have similar physicochemistry, and we strongly suspect that sevoflurane interacts with the same site.

**Table 2:** The photolabeled residues of integrin αIIbβ3 by azi-isoflurane.

Purified αIIbβ3 from platelets.		
	Sequence coverage	Photolabeled residues
α subunit	29.58%	N/A
β subunit	33.22%	K159

Recombinant full ectodomain αIIbβ3		
	Sequence coverage	Photolabeled residues
α subunit	40.41%	N/A
β subunit	40.93%	D158, K159

### D158A mutant reduced the activation of αIIbβ3

**Figure 8:**
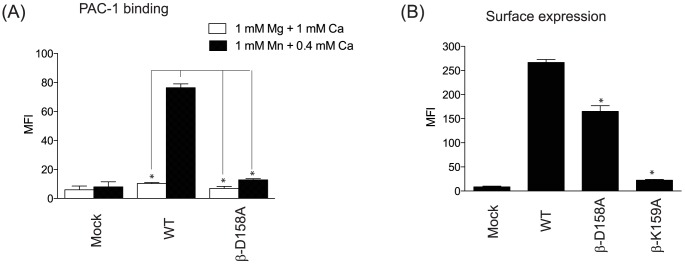
β3 mutants of adducted residues. (A) PAC-1 binding to mock, αIIbβ3 wild type or mutant in 1 mM Mg^2+^/Ca^2+^ or 1 mM Mn^2+^/0.4 mM Ca^2+^. MFI; mean fluorescence intensity. One-way analysis of variance with Tukey post hoc analysis was performed to compare different groups (excluding mock group). * denotes p<0.05 versus wild type, 1 mM Mn^2+^/0.4 mM Ca^2+^ group. (B) Surface expression of mock, αIIbβ3 wild-type or mutants probed by AP3 is shown. Data is shown as mean +/− S.D. of three independent experiments. One-way analysis of variance with Tukey post hoc analysis was performed (excluding mock group). * denotes p<0.05 versus wild type.

To confirm the functional role of the azi-isoflurane adducted residues (Asp-158 and Lys-159), we made β3-D158A and –K159A mutants to alter the chemical texture of this site. As shown in **Figure 8A**, β3-D158A mutant completely abolished the activation of αIIbβ3 integrin in activating conditions (1 mM Mn^2+^/0.4 mM Ca^2+^), indicating the importance of this residue. This was consistent with the previously reported results of the mutants of the other SyMBS forming residues [26]. The β3-K159A mutant significantly diminished the cell surface expression of αIIbβ3 (**Figure 8B**), suggesting that Lys-159 was a critical residue for expression rather than activation.

## Discussion

In this study, we demonstrated that isoflurane and sevoflurane, but not propofol, attenuated the activation of integrin αIIbβ3. Furthermore, the photolabeling experiment using azi-isoflurane suggested that isoflurane bound to the residues around the SyMBS of the I domain of the β subunit. That these two findings were linked was suggested by the mutagenesis experiments, which indicated the importance of this site for expression and activation.

With the appreciation of its profound effect on platelet aggregation, αIIbβ3 has been an attractive therapeutic target to prevent platelet aggregation in specific disease states. For example, peptides containing the RGD sequence competitively prevent αIIbβ3 from binding to its ligands [22], and have thus served as a basis for antagonist design [17]. Currently, abciximab (Reo-Pro, Eli Lilly, Indianapolis, IN), eptifibatide (Integrelin, Cor therapeutics, Cambridge, MA) and tirofiban (Aggrastat, Merck, Whitehouse Station, NJ) are approved for clinical usage to reduce ischemic events in patients with acute coronary syndrome undergoing percutaneous coronary intervention [45], [46]. When these drugs were developed, there was no structural information how these compounds interacted with αIIbβ3. Now we know that the majority of αIIbβ3 small molecule antagonists including eptifibatide and tirofiban bind to a small pocket on the top of the αIIbβ3 head formed by loops from the αIIb β-propeller and the βI domain [24]. These compounds interact with the MIDAS Mg^2+^ ion of the I domain via one of the oxygen atoms in the compound's Asp carboxyl or an equivalent carboxyl [24], [25]. The exception is abciximab, the β3 specific-7E3 Fab, which blocks ligand binding by binding to residues in the specificity determining loop (SDL) [47] (**Figure 7A**). Macromolecules such as fibrinogen recognize a rather larger area at the interface between the β-propeller domain of the αIIb subunit and the I domain of the β subunit, and interact with the β3 SDL and αIIb β-propeller loops. Therefore, blocking SDL wth abciximab prevents fibrinogen binding [24].

Surprisingly, the adducted residues of photolabeling experiments were located around the SyMBS, which was not at the binding pocket of the aforementioned αIIbβ3 small molecule antagonists and Fab. The SyMBS coordinates Ca^2+^ and allosterically activates integrins for ligand binding by stabilizing the MIDAS site [36]. The side chain carboxyl of β3-Glu-220 coordinates the SyMBS Ca^2+^ and MIDAS Mg^2+^ at the same time [36] (**Figure 7B**). Therefore, any alteration of residues surrounding the SyMBS could influence the orientation of MIDAS, and therefore αIIbβ3 activation. The result of β3-D158A mutant supported this idea. Also, the SyMBS coordinates with the SDL and disruption of this interaction resulted in impaired activation, as shown by blockade of ligand binding by abciximab [48]. Thus, allosteric inhibition of activation via binding to SyMBS is feasible. However, azi-isoflurane is structurally altered from isoflurane, and it is possible that the sites reported could be different from isoflurane binding site(s). However, the crystallographically proven identity of azi-isoflurane and isoflurane protein binding sites in our previous reports argues against this possibility. Co-crystallization of isoflurane with αIIbβ3 may answer this question in the future. Additionally, we cannot exclude the existence of other binding site(s) on regions of the protein that we were not able to detect using mass spectrometry.

Interestingly, we found the adducted residues only on the I domain of the β3 subunit in αIIbβ3 with two different preparations. In addition to the αIIb subunit, the β3 subunit couples with the αV subunit to form integrin αVβ3. The number of αVβ3 copies on platelets is small compared with that of αIIbβ3 [20], but αVβ3is highly expressed on endothelial cells. Both αIIbβ3 and αVβ3integrins bind to fibrinogen, but at different sites, forming a cooperative interaction between αIIbβ3 and αVβ3 that allows the platelet thrombus to be anchored on the endothelium through αVβ3 [49], [50]. It is possible that sevoflurane and isoflurane also impair the activation of αVβ3 as well to diminish the anchoring of platelets on the endothelium, which will be an additional effect to impair hemostasis by volatile anesthetics.

Clinical significance of functional alternation in αIIbβ3 is apparent from a familial bleeding disease, Glanzmann thrombasthenia. Bleeding in this disorder derives from the failure of platelet aggregation due to reduced or absent αIIbβ3 [51]. Therefore, the fact that sevoflurane and isoflurane directly modulate the activation of αIIbβ3 can be clinically significant. Our results are entirely consistent with this and with the previous clinical reports of endoscopic sinus surgeries. Although many studies have been performed in this surgical population, the numbers of patients enrolled in each study are not large (**Table 1**). Future clinical investigation will be extremely important, particularly on cases at high risk of bleeding such as scoliosis and vascular surgeries. Since blood products are not unlimited resources and not entirely risk-free, this is an important health care consideration. The choice of anesthetic drugs may need to be considered from hemostasis standpoint.

In conclusion, we have demonstrated that the inhalational anesthetics isoflurane and sevoflurane, not but the intravenous anesthetic propofol, impairs the activation of integrin αIIbβ3 via a direct novel allosteric mechanism.
